# Bifunctional HDAC Therapeutics: One Drug to Rule Them All?

**DOI:** 10.3390/molecules25194394

**Published:** 2020-09-24

**Authors:** Joshua P. Smalley, Shaun M. Cowley, James T. Hodgkinson

**Affiliations:** 1 Leicester Institute of Structural and Chemical Biology, School of Chemistry, University of Leicester, George Porter Building, University Road, Leicester LE1 7RH, UK; jps43@leicester.ac.uk; 2 Department of Molecular and Cell Biology, University of Leicester, Lancaster Road, Leicester LE1 9HN, UK; smc57@le.ac.uk

**Keywords:** histone deacetylase, epigenetics, dual inhibitor, hydroxamic acid, *o*-aminoanilide, class selective, complex selective, cancer, CoREST, PROTAC

## Abstract

Histone deacetylase (HDAC) enzymes play crucial roles in epigenetic gene expression and are an attractive therapeutic target. Five HDAC inhibitors have been approved for cancer treatment to date, however, clinical applications have been limited due to poor single-agent drug efficacy and side effects associated with a lack of HDAC isoform or complex selectivity. An emerging strategy aiming to address these limitations is the development of bifunctional HDAC therapeutics—single molecules comprising a HDAC inhibitor conjugated to another specificity targeting moiety. This review summarises the recent advancements in novel types of dual-targeting HDAC modulators, including proteolysis-targeting chimeras (PROTACs), with a focus on HDAC isoform and complex selectivity, and the future potential of such bifunctional molecules in achieving enhanced drug efficacy and therapeutic benefits in treating disease.

## 1. Introduction

**Figure 1:**
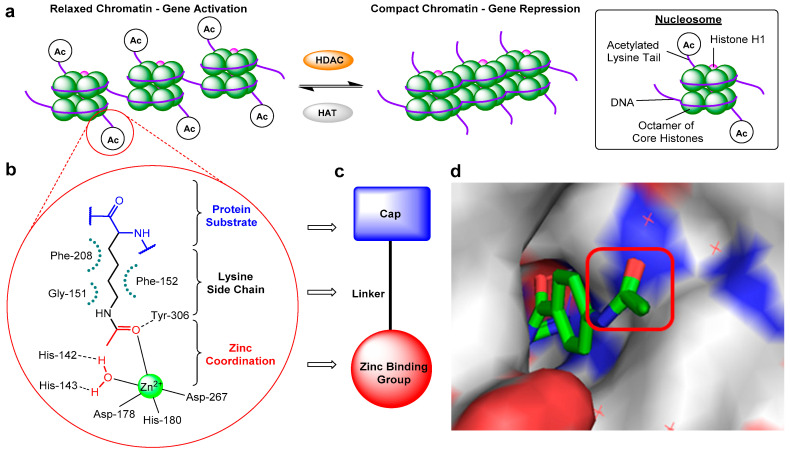
(**a**) Schematic diagram illustrating changes in chromatin structure due to histone deacetylase (HDAC)-catalysed deacetylation. (**b**) Representative HDAC–acetyl lysine substrate interactions in an active site. (**c**) Typical HDAC inhibitor design. (**d**) Crystal structure of *o*-aminoanilide HDAC inhibitor bound to HDAC2, highlighting the surface-exposed acetyl group and hence cap modification tolerance for dual inhibitor functionalisation (PDB 4LY1).

The reversible acetylation and deacetylation of protein substrates play critical roles in the regulation of epigenetic gene expression and other cellular processes [1,2,3,4]. These modifications are controlled by two opposing families of enzymes: histone acetyltransferases (HATs) and histone deacetylases (HDACs). In nucleosomal histone tail regions, HDACs catalyse the hydrolysis of *N*-ε-acetyl-l-lysine side chains to afford acetate and free l-lysine (Figure 1a), resulting in a more compacted chromatin structure which prevents transcription factors and RNA polymerase from accessing gene promoter regions—hence, HDACs are widely associated with gene repression. In addition to histones, HDACs are also responsible for the deacetylation of lysine residues in other proteins, including α-tubulin, heat shock protein 90 (Hsp90), as well as a variety of transcription factors and DNA repair proteins [5]. There are 18 different HDACs present in the human genome. These are divided into two distinct families based on their requirements for activity, as well as four classes based on their sequence homology [6,7]. The more widely explored family of HDACs is the zinc-dependent family, comprising of 11 isoforms divided into 3 classes: class-I (HDACs 1, 2, 3, and 8), class-II (further subdivided into IIa: HDACs 4, 5, 7, and 9, plus IIb: HDACs 6 and 10), and class-IV (HDAC11). The second family requires the cofactor NAD^+^ for activity as opposed to zinc and encompass the structurally and mechanistically unrelated class-III HDACs, also known as sirtuins (SIRT1-7).

Abnormal changes in HDAC expression and therefore the levels of deacetylation have been associated with a range of diseases, including many cancers [8]. For example, HDACs have been shown to influence the expression of numerous genes in both cancer initiation and progression, plus play an essential role in many signalling pathways that promote malignant cell survival [9,10]. Consequently, pharmacological targeting of HDACs has emerged as an important therapeutic area of research, with the discovery of HDAC inhibitors for the treatment of cancers such as leukaemias and myelodysplastic disorders [11], as well as Alzheimer’s disease [12], Huntington’s disease [13], muscular atrophy [14] and Friedrich’s ataxia [15].

Despite mediating the acetylation status of various proteins, the zinc-dependent HDACs possess a mostly conserved catalytic active site (Figure 1b) [16,17], hence the majority of synthesised HDAC inhibitors encompass a generic three-part “cap-linker-zinc binding group” pharmacophore model (Figure 1c) [18]. The crucial aspect of the design is the zinc chelator, which functions by inserting into the HDAC active site and binding zinc at the bottom of the enzyme pocket, usually in a bidentate approach. Next is the linker section, which mimics the substrate lysine side chain by fitting the 11 Å tube-like channel leading to the zinc ion, plus maintaining the multiple hydrophobic interactions within the active site. The final component is the hydrophobic “cap” at the end of the linker, which often contains aromatic moieties that interact with residues near the outer region of the active site or on the protein external surface. The terminal cap points out towards solvent and is routinely optimised to afford inhibitors with HDAC isoform selectivity (Figure 1d).

**Figure 2:**
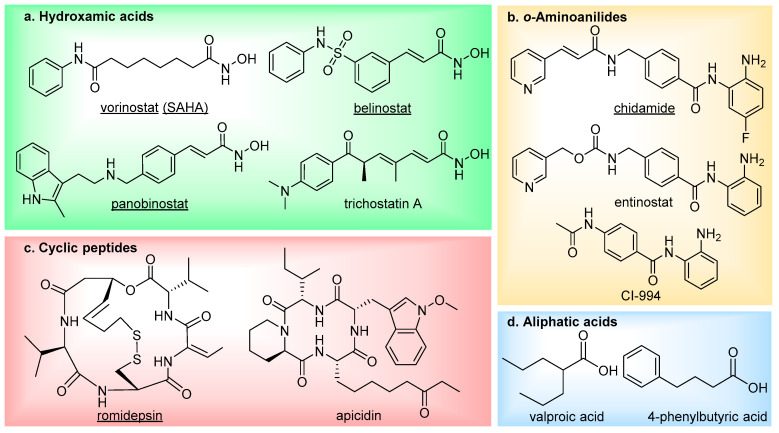
The main four classes of HDAC inhibitors, including FDA-approved drugs (underlined).

There are currently four main structural classes of HDAC inhibitors; hydroxamic acids, *ortho*-aminoanilides, cyclic peptides and aliphatic acids (Figure 2) [19]. The inhibitors are categorised based on their zinc-binding group; however, many structural similarities are seen between the classes. The hydroxamic acids are generally broad-range inhibitors (target HDACs 1–11) and potent at low nM concentrations, causing cell cycle arrest and apoptosis in cultured cells [20]. Conversely, the aliphatic acids are much weaker inhibitors, although, they do exhibit modest class-I HDAC selectivity. The *o*-aminoanilides, also referred to as benzamides, exhibit high class-I selectivity, as do the cyclic peptides [21].

To date, five HDAC inhibitors have been approved for cancer treatment-vorinostat and romidepsin to treat refractory cutaneous T-cell lymphoma [22,23], panobinostat for patients with multiple myeloma [24], plus belinostat and chidamide for the treatment of relapsed or refractory peripheral T-cell lymphoma [25,26]. These and additional HDAC inhibitors have also entered clinical trials for the treatment of other types of cancer [27] (NCT00138203, NCT00077194, NCT00451035, NCT00828854, NCT02236195), as well as non-oncology indications such as neurodegenerative diseases (NCT02124083, NCT00212316) and epilepsy (NCT03894826). Although first generation HDAC inhibitors have demonstrated therapeutic significance, especially in treating hematologic malignancies, there has been limitations both in their selectivity and efficacy which has restricted further clinical applications. They frequently suffer from poor HDAC isoform selectivity, resulting in high toxicity and severe side effects [28,29,30,31,32,33]. In addition to this, HDAC inhibitors have also been shown to be less potent against solid tumours, thus hindering employment in cancer treatment [34,35,36,37,38]. Current investigations to overcome these challenges are split into the development of three main branches of HDAC inhibitor: isoform-selective HDAC inhibitors, complex-selective HDAC inhibitors (in the case of class-I HDACs), and bi- (or multi-) functional HDAC inhibitors. The latter have received significant interest over the last decade, with an increasing number of applications.

## 2. Dual HDAC Inhibitors

The majority of the current drug discovery landscape has centred on single-agent single-target therapeutics, often exhibiting impressive selectivity and potency. However, for multifactorial disease types such as cancer or neurological disorders, single target drugs alone are often unable to provide effective treatment and can be more susceptible to drug resistance [39,40]. One approach to offer more durable and targeted disease control has been through the application of combinational drug treatment, often termed “drug cocktails”, involving the simultaneous administration of two or more agents [41,42]. Despite proven therapeutic relevance to this strategy and its common wide use, it does have a number of limitations including; poor patient compliance, a complex pharmacokinetic profile, unpredictable drug–drug interactions, formulation problems, high development costs and complicated dose–schedule regimes [43]. In order to overcome these drawbacks, there has been a growing interest towards conjugating two distinct pharmacophores together to create single molecule, dual-targeting therapeutics [44,45,46]. This polypharmacological approach allows one molecule to modulate multiple targets simultaneously and often synergistically in order to achieve a greater therapeutic advantage over single-targeting agents [47,48]. Compared to drug cocktails, it also offers the advantage of a more predictable pharmacokinetic profile, modulation of drug resistance, a less complex regulatory process, as well as reduced patient compliance difficulties, clinical trial costs and drug-drug interactions [49,50]. Dual inhibitors are thus very promising candidates for future therapeutics.

Designing dual inhibitors presents a number of significant challenges. The drug must retain effective binding to two or more target proteins while also maintaining desirable physiochemical and pharmacokinetic properties, particularly for the development of oral drugs. Consequently, target selection and molecule design are key aspects in dual inhibitor development. One of the most attractive and well-suited targets to this technology are HDACs, due to their implications in complex diseases such as cancer [7], neurodegenerative conditions [51], asthma [52], and diabetes [53], which possess multiple pathophysiological mechanisms. Bifunctional HDAC inhibitor therapeutics have the potential to simultaneously block two disease-related targets and thereby offer an improved and more comprehensive treatment over single-targeting agents. There have been numerous reports of HDAC inhibitors demonstrating synergistic effects in combination with other small-molecule drugs, thus enhancing therapeutic benefit [54,55]. In cancer treatment, HDAC inhibitors containing drug cocktails have shown promising antitumour activities [56]. A combination of vorinostat with the endocrine therapy drug tamoxifen was effective in reversing hormone resistance and caused tumour regression or prolonged disease stabilisation in 40% of patients [57] (NCT00365599). In another study, panobinostat was combined with bortezomib and dexamethasone to treat patients with relapsed multiple myeloma, which resulted in a significant increase in progression-free survival versus placebo plus bortezomib and dexamethasone (NCT01023308). With regards to molecule design, due to the large hydrophobic region at the surface rim of the HDAC active site, the cap section of HDAC inhibitors can tolerate extensive structure variation. This is exemplified by the FDA approved HDAC inhibitor romidepsin—a bicyclic peptide whose “cap” consists of the majority of its structure and is very different to the other clinically approved inhibitors but maintains potency.

**Figure 3:**
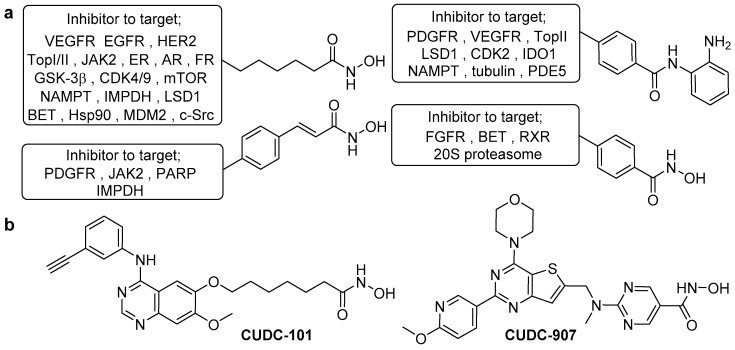
(**a**) The most common HDAC inhibitor scaffolds used in dual HDAC inhibitor design to date. Accompanying each are the relevant protein targets for which a compatible inhibitor has been conjugated to this HDAC inhibitor fragment for the development of a successful and potent dual-targeting inhibitor. (**b**) Two hydroxamic acid-based HDAC/kinase dual inhibitors in clinical trials.

In general, HDAC dual inhibitors are developed through a pharmacophore fusion approach. Their structure is an elaboration of single-targeting HDAC inhibitors (Figure 3a), with the cap incorporating the inhibitor for the secondary target. Over the past decade, there has been an explosion of reported dual HDAC inhibitors, achieving simultaneous blockade of HDAC and a variety of targets including kinases, topoisomerases, hormone receptors, plus other epigenetic regulators [58,59]. The therapeutic importance of such compounds has been demonstrated by the progression of HDAC/kinase dual inhibitors into clinical trials for cancer treatment (Figure 3b)—CUDC-101 (HDAC/EGFR inhibitor) and CUDC-907 (HDAC/PI3K inhibitor) [60,61,62,63,64]. In this perspective, we will focus on dual functionalised HDAC inhibitors that exhibit class, isoform and complex selectivity.

## 3. Class-I HDAC Dual Inhibitors

**Figure 4:**
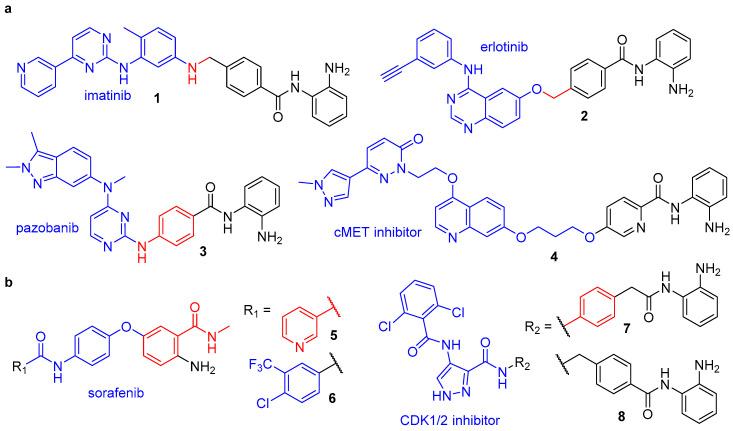
Class-I-selective HDAC dual inhibitors targeting (**a**) receptor tyrosine kinases and (**b**) other kinases. Components: kinase inhibitor (blue), HDAC inhibitor (black) and shared structural components (red).

The *o*-aminoanilide structural class of HDAC inhibitors is principally associated with selective targeting of class-I HDAC isoforms, in particular HDACs 1,2 and 3 [65]. This characteristic selectivity is observed because HDACs 1,2 and 3 possess an additional 14 Å wide cavity in the active site termed the foot pocket, which more favourably accommodates the 7-membered ring chelate formed by the larger *o*-aminoanilide modality [66,67]. Further exploration of the internal cavity has produced inhibitors containing various aryl substituents on the *o*-aminoanilide warhead, which have achieved increased isoform selectivity and potency [68,69,70,71], plus more recently enabled complex-specific inhibition [72]. Conjugation of *o*-aminoanilide-based HDAC inhibitors with another specificity-targeting inhibitor has been employed to afford numerous class-I HDAC-selective dual inhibitors, with kinases being a frequently investigated secondary target (Figure 4).

### 3.1. Class-I HDAC/Kinase Dual Inhibitors

Receptor tyrosine kinases (RTKs) are a family of cell-surface receptors that play a crucial role in the processes of regulating cell proliferation, differentiation, and survival [73]. Their dysregulation has been shown to be a key mediator of cancer progression, and, as such, RTK inhibitors have emerged as important therapeutic agents. Two clinically proven examples include the Abl, PDGFRβ and Kit inhibitor imatinib and the EGFR/HER2 inhibitor erlotinib, both of which have demonstrated additive anti-cancer effects when used in combination with HDAC inhibitors [74,75,76,77]. In separate works by Mahboobi et al., imatinib and erlotinib were equipped with a variety of *o*-aminoanilide and hydroxamic acid HDAC inhibitor warheads to afford the dual RTK/HDAC inhibitors **1** and **2** [78,79]. These hybrids both incorporated the HDAC inhibitor entinostat into their design and were shown to display class-I HDAC isoform selectivity—with potent inhibition of HDAC1 (IC_50_ = 0.208 and 0.041 µM for **1** and **2,** respectively) and inactivity against HDAC6 (IC_50_ = >32 µM for **1** and **2**). Compound **2** was also screened against other class-I isoforms which revealed a greater than 10-fold selectivity for HDAC1 over HDAC3 (IC_50_ = 0.55 µM) and no inhibition of HDAC8 (IC_50_ = >32 µM). Concerning RTK inhibition, **1** demonstrated the most efficient inhibition of Abl kinase (IC_50_ = 2.0 µM) and the imatinib-resistant Abl^T315I^ mutant (IC_50_ = 1.1 µM) of all the synthesised hybrids, with efficient cellular inhibition of PDGFR (IC_50_ = 2.7 µM) and cytotoxicity towards EOL-1 cells (IC_50_ = 0.1 µM). Compound **2** showed strong inhibitory activity towards EGFR and HER2, with IC_50_ values of 0.078 and 0.066 µM, respectively, along with strong cytotoxicity against a panel of tumour cell lines.

In a similar study, the VEGFR inhibitor pazopanib was also fused with entinostat to afford dual inhibitor **3** [80]. Pazopanib is approved for the treatment of advanced renal cell carcinoma and soft tissue sarcoma, however, like HDAC inhibitors, when used solely, without drug combinations, its efficacy is limited by the development of acquired drug resistance [81,82,83]. Interestingly, combinations of HDAC inhibitors and pazopanib have demonstrated synergistic therapeutic effects in addition to a reversal of drug resistance, thus conjugating these molecules in a multitargeted drug approach offers significant therapeutic potential [84,85]. In vitro assays revealed **3** possessed an analogous HDAC inhibition profile to entinostat, with IC_50_ values of 0.59, 0.91 and 0.43 μM, respectively, for HDACs 1–3, and selectivity for these class-I HDACs over HDAC6/8 isoforms [80]. Equally, VEGFR-2 inhibition for **3** (IC_50_ = 37 nM) was unchanged from pazopanib (IC_50_ = 34 nM). Evaluation of antiproliferative activities against a HT-29 solid tumour cell line showed **3** to be more potent (IC_50_ = 1.07 μM) than both entinostat (IC_50_ = 3.10 μM) and the hydroxamic acid-based inhibitor vorinostat (IC_50_ = 1.51 μM). In a subsequent HT-29 xenograft model, **3** displayed considerable in vivo antitumour efficacy, plus investigation of its pharmacokinetic profile revealed an impressive oral bioavailability of 72% in Sprague-Dawley (SD) rats [80].

Continuing the development of RTK/HDAC dual inhibitors, in 2017, Lu et al. reported the first example to target the RTK c-Met [86]. Herein, a selective c-Met inhibitor was fused with HDAC inhibitor pharmacophores to afford a series of hybrid molecules. Previous work by the group revealed the substituent at C7 of the quinoline moiety in the c-Met inhibitor protrudes into the solvent-exposed region of the protein and can tolerate side chain modification with no effect on inhibition and thus served as an attractive position for functionalisation to the HDAC inhibitor [87]. Enzymatic assays on the preliminary library of compounds revealed *o*-aminoanilide as the optimal zinc binding group (ZBG) for design of dual HDAC/c-Met inhibitors. A subsequent structure activity relationship (SAR) study investigating alternative linkers to equip the *o*-aminoanilide head group uncovered **4**, which contained an alkyloxypyridine linker, as the most potent dual inhibitor. Hybrid **4** demonstrated nanomolar inhibition of both c-Met (IC_50_ = 0.71 nM) and HDAC1 (IC_50_ = 38 nM) as well as effective antiproliferative activities against EBC-1 (IC_50_ = 0.058 µM) and HCT116 (IC_50_ = 1.3 µM) tumour cell lines, in each case displaying enhanced potency over reference class-I HDAC inhibitor chidamide and the parent c-Met inhibitor. In EBC-1 cells, **4** inhibited phosphorylation of c-Met and its key downstream signalling proteins, as well as increased H3 acetylation (Ac-H3) levels and p21 expression. Importantly, tubulin acetylation levels (Ac-tub, mediated by HDAC6) were unaffected by treatment with **4**, signifying class-I HDAC-selective inhibition.

Other therapeutically relevant kinases to have been targeted via dual HDAC inhibitor conjugates include the Raf proteins, central components of the MAPK/ERK pathway which regulates cell proliferation. Geng et al. reported the first potent dual Raf/HDAC inhibitors, which feature an unusual *o*-aminoanilide ZBG that differs from the conventional scaffold seen in HDAC inhibitors such as entinostat [88]. The design of these hybrids was built upon previous work within the group, wherein a novel class of diphenyl ether HDAC inhibitors were discovered from a high-throughput screening investigation. The lead compound **5** possessed a moderate IC_50_ of 5.23 µM for HDAC1 and contained the *o*-aminoanilide and pyridyl unit seen in the HDAC inhibitor chidamide. However, more interestingly, it also possessed an acylaminophenoxybenzamide scaffold similar to that of the FDA approved Raf kinase inhibitor sorafenib—a drug which in combination with HDAC inhibitors has shown synergistic effects in cancer treatment [89]. A subsequent kinase activity assay revealed **5** moderately inhibited BRaf^V600E^ with an IC_50_ of 1.78 µM, thus functioning as a dual inhibitor. In this later work, **5** was optimised via a SAR study to develop **6**, which incorporated the 4-chloro-3-(trifluoromethyl)phenyl moiety also seen in sorafenib. Enzyme assays with **6** revealed potent inhibition against BRaf^V600E^ (IC_50_ = 0.073 µM) and HDAC1 (1.17 µM), significant enhancements in potency compared to **5** and equivalent BRaf^V600E^ inhibition to sorafenib (IC_50_ = 0.065 µM). Investigation into enzyme isoform selectivity revealed **6** to be a pan-inhibitor like sorafenib for Rafs A–C but exhibited selectivity for HDAC1 (HDAC2 and HDAC3 not investigated), with no inhibitory activity observed against HDAC6 or HDAC8. The hybrid also showed proficient antiproliferative activities against a variety of cancer cell lines, with notably greater potency against HepG2 (IC_50_ = 1.33 µM) and MDA-MB-468 (IC_50_ = 0.57 µM) cell lines than the reference inhibitors sorafenib (IC_50_ = 3.04 and 12.17 µM) and chidamide (IC_50_ = 4.89 and 2.65 µM), demonstrating the benefit of multi-target therapeutics over single-target agents.

A comprehensive study by Cheng et al. recently reported a series of novel HDAC/cyclin-dependent kinase (CDK) dual inhibitors, combining the main pharmacophore of a known CDK1/2-selective inhibitor with hydroxamic acid and *o*-aminoanilide moieties [90]. Antiproliferative activity and enzyme inhibition assays revealed the *o*-aminoanilide-based compounds to be significantly more potent than those containing the hydroxamic acid ZBG. Since both HDAC and CDK inhibitors have proven antiproliferative effects, the most promising dual inhibitors were further examined for antiproliferative activity against a panel of five human cancer cell lines. Hybrids **7** and **8** were found to exhibit the greatest potency overall, with IC_50_ values of 1.45 and 0.71 µM, respectively, against HCT116 cells—more potent than both the parent CDK inhibitor (IC_50_ = 1.96 µM) and the reference HDAC inhibitor chidamide (IC_50_ = 2.77 µM). Enhanced efficacy over the reference inhibitors was even more pronounced in subsequent in vitro inhibition assays (Table 1), which revealed **7** and **8** to possess significant inhibitory activity and isoform selectivity for HDAC2 (IC_50_ = 0.24 and 0.25 nM) and CDK2 (IC_50_ = 0.56 and 0.30 nM). In particular, compound **7** exhibited 1000-fold selectivity for HDAC2 over HDAC1, despite these two enzymes sharing an 86% sequence homology, plus > 4000-fold selectivity over the other class-I HDACs (HDAC 3 and 8) and the class-II isoform HDAC 6. Both **7** and **8** displayed greater than 20-fold selectivity for CDK2 over CDK1, with negligible inhibition of CDKs 4, 6 and 7. Additional studies revealed **7** and **8** could promote apoptosis in a variety of cancer cell lines, later supplemented with the evidence that they enhanced intracellular ROS levels in A375 cells—a key activator of cell death. Progressing investigation to an in vivo setting revealed hybrid **8** to have more favourable pharmacokinetic properties, including a bioavailability of 63.6% in ICR mice, as well as efficient antitumour activity in an HCT116 xenograft model [90].

### 3.2. Class-I HDAC/Non-Kinase Dual Inhibitors

**Figure 5:**
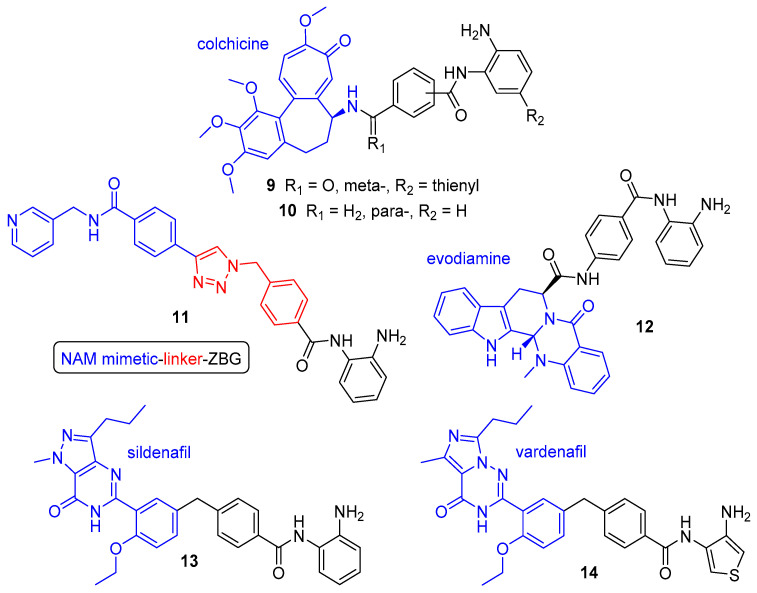
Non-kinase-targeting class-I-selective dual HDAC inhibitors. Components: HDAC inhibitor (black) and moiety for inhibition of second protein target (blue).

In addition to kinases, there a variety of other protein targets that have been addressed using class-I-selective dual HDAC inhibitors (Figure 5). The natural product colchicine is a tubulin inhibitor approved for the treatment of gout, however, its ability to disrupt tubulin polymerisation also serves as a useful anticancer effect owing to the critical role of microtubule assembly in cell division and motility [91]. HDAC inhibitors have been shown to display synergistic antitumour activities in combination with tubulin inhibitors [54,92], prompting the development of HDAC/tubulin dual inhibitors as a means of achieving improved therapeutic effect. Zhang and co-workers reported the first examples, conjugating colchicine to hydroxamic acid and *o*-aminoanilide moieties [93,94]. Of the *o*-aminoanilide-based hybrids, compounds **9** and **10** emerged as the most promising. Containing a thienyl substituent, **9** displayed potent class-I HDAC inhibition, with selectivity for HDAC2 (IC_50_ = 0.19 µM) over HDACs 1 and 3 (IC_50_ = 1.50 and 1.49 µM, respectively). It also displayed comparable tubulin inhibition and antiproliferative activity to colchicine. Dual inhibitor **10**, despite exhibiting similar tubulin inhibition and far weaker inhibition of class-I HDACs (IC_50_ = 12.50, 6.73 and 11.23 µM, respectively, for HDACs 1–3), was found to have equal or superior antiproliferative activity (IC_50_ = 2–106 nM) across an entire panel of 11 human cancer cell lines than both **9** and colchicine [94].

The enzyme nicotinamide phosphoribosyltransferase (NAMPT) has also been successfully combined with HDAC inhibition to deliver potent NAMPT/HDAC dual inhibitors. NAMPT is a rate-limiting enzyme which regulates the intracellular levels of nicotinamide adenine dinucleotide (NAD), a central cofactor for cellular metabolism. It is overexpressed in several cancers, supplying high levels of NAD to fuel cell proliferation [95]. NAMPT inhibitors have emerged as promising drug candidates to block cancer cell proliferation and induce apoptosis, however, alike HDAC inhibitors, they are hampered by dose-limiting toxicities when used in isolation without drug combinations [96]. Interestingly, both types of inhibitor also possess a similar structural design, consisting of three fragments—a head group (ZBG for HDAC inhibitors and a nicotinamide (NAM) mimetic for NAMPT inhibitors), a linker and a hydrophobic cap [97]. In an elegant piece of work by Dong et al., the two pharmacophore models were fused to afford a series of dual-targeting inhibitors comprising a “ZBG-linker–NAM mimetic” design [98]. These hybrids utilised the main scaffold of the *o*-aminoanilide-based HDAC inhibitor CI-994 and focused on SAR optimisation of both the linker and NAM mimetic components. In vitro assays revealed that the hybrids containing 1,2,3-triazole rings were markedly more potent than any of the other synthesised compounds—an observation consistent with related reports on NAMPT inhibitors [99]. Of these, compound **11** emerged as the most potent dual inhibitor, with balanced activities against NAMPT (IC_50_ = 31 nM) and HDAC1 (IC_50_ = 55 nM), plus efficient antitumour activity against a variety of cell lines. Investigation with other HDAC isoforms revealed comparable inhibition for HDAC 2 (IC_50_ = 75 nM) and selectivity over other class-I and -II HDACs (IC_50_ = 1.87 µM for HDAC3 and >100 µM HDACs 4,6 and 8). Studies in the HCT116 cell line showed **11** could effectively increase histone acetylation levels, reduce cellular levels of NAD, and consequently induce cell cycle arrest, apoptosis and autophagy. Subsequent treatment of **11** in a HCT116 xenograft model demonstrated superior in vivo antitumour efficacy over reference drugs vorinostat and FK866 (NAMPT inhibitor) [98].

In addition to compound **11**, the Sheng group also fused CI-994 with the topoisomerase II (TopII) inhibitor evodiamine to afford dual inhibitor **12** [100]. TopII is as DNA topoisomerase which relaxes supercoiled DNA via double-strand cleavage and plays crucial roles in transcription and replication. The action of TopII inhibitors is to stabilise the DNA-enzyme cleavable complex via intercalation between the DNA base pairs, thus generating enzyme-mediated DNA damage and initiating cell death [101]. TopII inhibitors have been successfully utilised in combination with HDAC inhibitors for potentiation of activity, as the hyperacetylation of histone lysine tails induces chromatin relaxation and increases access to DNA [102]. Whilst TopII has been subject to a variety of examples of potent TopII/HDAC dual inhibitors, the majority contain a hydroxamic acid ZBG and consequently exhibit pan-inhibition of HDAC enzymes [103,104,105]. Compound **12** however displayed remarkable selectivity for HDAC1 (IC_50_ = 0.16 µM) against a broad panel of HDAC isoforms, with very little inhibition seen for HDACs 2,3 and 10 (IC_50_ = 10, 27 and 5.5 µM, respectively) and no inhibition for HDACs 4,6,8 or 11. Selective TopII inhibition was investigated via a TopI- and TopII-mediated DNA relaxation assay, with **12** demonstrating high levels of supercoiled DNA when treated with TopII but no supercoiled DNA bands when treated with TopI. Evaluation of in vivo antitumour activity with **12** (150 mg/kg, bid) in a HCT116 xenograft model led to a tumour growth inhibition (TGI) of 75.2%—much enhanced over equivalent treatment with reference drugs vorinostat (TGI = 40.8%) and evodiamine (TGI = 45.5%), plus their combination (TGI = 54.5%).

Further to their primary use as anticancer agents, HDAC inhibitors have also proven to be important therapeutic drugs for a range of other diseases, including neurodegenerative disorders such as Alzheimer’s disease (AD) [106]. Another class of inhibitors to exert anti-AD effects are phosphodiesterase (PDE) inhibitors, which have been shown to effectively restore memory function through enhancing levels of cyclic adenosine monophosphate (cAMP) and cyclic guanosine monophosphate (cGMP)—critical second messengers for neuronal signalling in the nitric oxide/cGMP/cAMP-responsive-element-binding (CREB) pathway [107]. Of these, PDE5 inhibitors such as sildenafil and vardenafil act to increase cGMP concentrations, activating the phosphorylation of CREB and thereby enhancing synaptic function and memory in AD [108]. In a multitargeted approach to treat AD, these two inhibitors were attached using a range of linkers to an *o*-aminoanilide (or thiophene equivalent) ZBG to synthesise a library of dual PDE5/HDAC inhibitors [109]. Sildenafil-based compound **13** and vardenafil-based compound **14** were identified as two of the most potent hybrids in the series, with strong inhibition of PDE5 (IC_50_ = 114 and 26 nM) and class-I HDACs (**13**, IC_50_ = 180, 660 and 777 nM for HDACs 1,2 and 3 at 4 h; **14**, IC_50_ = 97 and 406 nM for HDACs 1 and 2 at 4 h (HDAC3 not tested)). Given the slow binding kinetics associated with *o*-aminoanilide HDAC inhibitors [110,111], during investigation of HDAC inhibitory activity, the dual inhibitors were assayed after both a 30 min and 4 h preincubation time to prevent an underestimation of potency and hence afford accurate IC_50_ values with correct selectivity profiles. This proved valuable, with notably weaker inhibition observed in many cases for the shorter preincubation. Compound **13** in particular displayed significant time-dependent inhibition, with IC_50_ values increasing to 673 nM for HDAC1 and >20 µM for both HDAC2 and HDAC3 when preincubated for 30 min—thus providing a misleading high selectivity for HDAC1 at the shorter time period. As expected, **13** and **14** were inactive towards HDAC6 at either incubation time, thus inferring class-I selectivity. Investigation of dual inhibitor-induced cellular responses found **13** to display the greatest increase in histone acetylation (AcH3K9) and CREB phosphorylation levels, along with the highest permeability in a parallel artificial membrane permeability (PAMPA) assay. Dual-target engagement of **13** was confirmed in vivo with evidence of some partial restoration of memory function in a mouse model of AD, however, further optimisation is required.

### 3.3. HDAC8-Selective Dual Inhibitors

**Figure 6:**
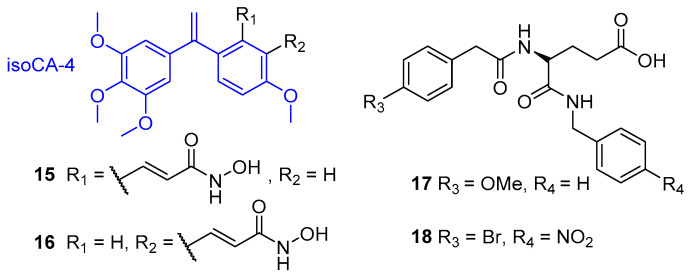
HDAC8-selective dual HDAC inhibitors.

Unlike HDACs 1,2 and 3, the class-I isoform HDAC8 does not exist as part of a multi-subunit co-repressor complex in vivo and is instead fully active in isolation as a transcription repressor [112,113]. HDAC8 possesses a unique active site structure and is therefore sensitive to a different inhibitor structural profile compared to the other class-I enzymes—as displayed with minimal inhibition activity against *o*-aminoanilide-based HDAC inhibitors. There have however been many HDAC8-selective inhibitors reported to date [114], plus a multi-targeted approach has also been implemented to discover dual inhibitors with selectivity towards HDAC8 (Figure 6).

The natural product combretastatin A-4 (CA-4) is an inhibitor of tubulin polymerisation which functions as a vascular disrupting agent to target tumour blood cells and induce tumour necrosis [115]. It is structurally related to colchicine (employed in hybrids **9** and **10**) and has been utilised in combination with chemotherapy and antiangiogenic drugs for enhanced and sustained therapeutic effects [116,117]. A CA-4 analogue termed *iso*combretastatin A-4 (*iso*CA-4) was developed by the Alami group and equipped with broad spectrum HDAC inhibitors vorinostat and belinostat to afford a series of dual-targeting agents [118]. In vitro assays revealed compounds **15** and **16** as the most potent dual inhibitors, both of which contain the olefinic linker derived from belinostat. These two hybrids exhibited the best antiproliferative activities against HCT116 cells (**15** IC_50_ = 8 nM, **16** IC_50_ = 1.5 nM) and efficient inhibition of tubulin polymerisation (**15** IC_50_ = 2.1 µM, **16** IC_50_ = 1.6 µM). Inhibition profiling against HDAC isoforms 1–11 was performed to establish selectivity, with the dual inhibitors tested across the panel of HDACs at 10 µM. Compounds with inhibition >50% against an HDAC were considered to represent significant effects against that isoform. In this study, **15** and **16** demonstrated selective and near complete inhibition of HDAC8 (99% and 95%, respectively), with moderate inhibition of HDAC6 and HDAC11 (up to 65%) and light (<50%) or no inhibition against all of the other HDACs. Subsequent in vitro screening against the significant isoforms revealed high potency and selectivity for **15** and **16** towards HDAC8 (IC_50_ = 0.06 and 0.34 µM, respectively) over HDAC6 (IC_50_ = 5 and 15 µM) and HDAC11 (IC_50_ = 8 and 10 µM). Cytotoxicity studies against a panel of cancer cell lines revealed **16** to exhibit higher antiproliferative activity than *iso*CA-4, trichostatin A and the HDAC8-selective inhibitor PCI-34051 for each of the 10 cell lines used. Further investigation showed **16** could induce cell cycle arrest at the G_2_/M phase in cancer cells at low nanomolar concentrations via disruption of microtubule assembly.

In a novel approach by Halder and co-workers, pharmacophore mapping and molecular docking techniques were employed to design a series of MMP2/HDAC8 dual inhibitors [119]. Matrix metalloproteinases (MMPs) are a family of zinc-dependent enzymes responsible for the degradation of extracellular matrix proteins and who play crucial roles in physiological processes such as wound healing, tissue remodelling, angiogenesis and apoptosis [120]. Their dysregulation is associated with numerous pathological conditions and cancers, with the MMP2 isoform intrinsically involved in cancer invasion and metastasis [121]. Interestingly, the influence of MMP2 in such events has been shown to be suppressed by HDAC inhibitors, through upregulation of reversion-inducing-cysteine-rich protein with kazal motifs (RECK) [122], thus, dual targeting of MMP2 and HDACs offers synergistical therapeutic potential. MMP2 inhibitors consist of two components: a ZBG to bind the catalytic zinc ion and a small-molecule or peptidomimetic backbone which interacts noncovalently with specific hydrophobic pockets neighbouring the zinc-binding site to afford selectivity [123]. Following a previous molecular modelling study to understand the structural and physiochemical requirements of selective MMP2 inhibitors, the group sought to modify suitable examples via pharmacophore mapping to retain MMP2 inhibition and attain inhibitory activity towards HDAC8—exploiting the unique structural features in its active site [119,124]. The initially designed compound, **17**, was synthesised and found to possess strong inhibition of HDAC8 (IC_50_ = 3.8 µM) but weak inhibition of MMP2 (IC_50_ = 83.7 µM). Subsequent molecular docking with MMP2 (PDB: 1HOV) [125] was utilised in order to optimise **17** and improve MMP2 inhibition, affording a series of analogues. The most promising dual inhibitor was **18**, with IC_50_ values of 2.9 µM and 6.4 µM, respectively, for HDAC8 and MMP2. Hybrid **18** demonstrated intra-class selectivity, with no inhibitory activity against HDAC1 or HDAC2 (IC_50_ = >100 µM). Additionally, **18** displayed the most prominent anti-migratory effect, reduction in cell invasion (38–42%) and reduction in MMP2 expression (40%) of all assayed compounds. Molecular dynamics simulation revealed the selective HDAC8 inhibition of **18** may be a result of interactions with amino acid residues present in the internal ”acetate release channel”—of which is much smaller in HDAC8 than other HDACs [126].

## 4. Class-II HDAC Dual Inhibitors

**Figure 7:**
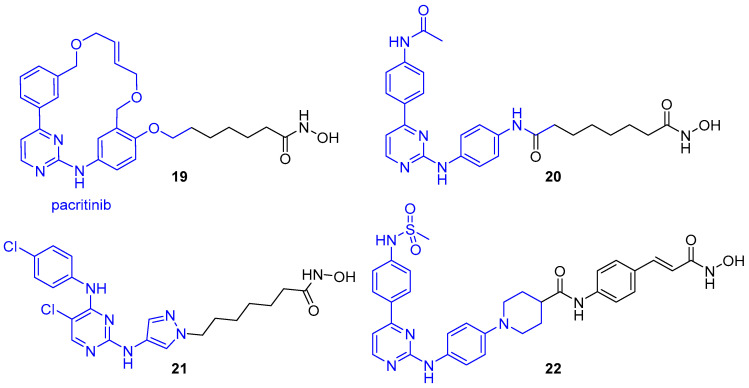
Janus kinases (JAK)-targeting HDAC6-selective dual inhibitors. Components: JAK inhibitor (blue), HDAC inhibitor (black).

**Figure 8:**
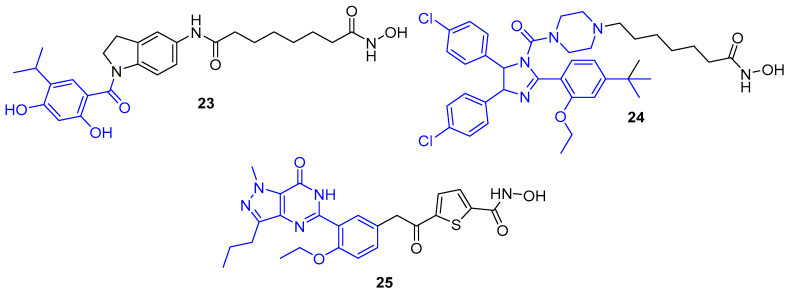
Other HDAC6-selective dual inhibitors. Components: HDAC inhibitor (black) and moiety for inhibition of secondary protein target (blue).

Hydroxamic acid-based HDAC inhibitors such as vorinostat and belinostat are those most frequently employed in the design of novel dual HDAC inhibitors. These inhibitors potently target both class-I and -II HDAC isoforms, with this pan-inhibition profile frequently conserved in the resulting hybrids. Consequently, whereas the *o*-aminoanilide motif is a proficient means of conferring class-I HDAC selectivity, achieving class-II-selective HDAC inhibition is not as straightforward or prevalent. In some cases, however, conjugation of a non-selective HDAC inhibitor with an inhibitor for a secondary target has afforded dual-targeted agents with impressive class-II selectivity, in particular towards HDAC6 (Figure 7 and Figure 8). This isoform selectivity parallels that of reported class-II HDAC mono inhibitors, of which only HDAC6 examples have been synthesised to date. HDAC6 is a unique member of the HDAC family as it contains two catalytic domains (CD1 and CD2) and a ubiquitin binding domain [127]. CD1 and CD2 are highly conserved, however, CD2 exhibits broad-specificity catalytic activity and is responsible for tubulin deacetylation [128]. Until 2016, with no reported HDAC6 crystal structure, the selectivity of HDAC6 inhibitors was frequently achieved through incorporation and optimisation of bulky or conformationally constrained hydrophobic caps to exploit the wider rim of the HDAC6 binding pocket [129,130,131]. Recent crystal structure determinations have provided a greater insight into the structural basis of HDAC6 inhibition and revealed the unique structural features of the enzyme active site which accommodate the short linker and bulky, rigid capping groups of HDAC6-selective inhibitors [132,133]. The significance of the cap to afford HDAC6 selectivity likely explains the acquired HDAC6 selectivity seen for some dual inhibitors, wherein HDAC6 more favourably accommodates the now larger cap possessed by the HDAC inhibitor following introduction of the secondary target inhibitor. The emergence of HDAC6 crystal structures paves the way for an emphasis on structure-based design over ligand-based design in the development of future HDAC6-selective mono and dual inhibitors.

### 4.1. JAK-Targeting HDAC6-Selective Dual Inhibitors

The most widely reported group of HDAC6-selective dual inhibitors concurrently target Janus kinases (JAKs) via conjugation of a JAK inhibitor with a hydroxamic acid ZBG warhead (Figure 7). The Janus kinases (JAK1, JAK2, JAK3 and TYK2) are a family of intracellular nonreceptor tyrosine kinases, which via activation from cytokines initiate the phosphorylation and dimerisation of STAT (signal transducers and activators of transcription) proteins for subsequent translocation to the nucleus and stimulation of gene transcription [134,135]. Via the JAK/STAT signalling pathway, JAKs control a range of important cellular events and immune responses, however, their dysregulation has been implicated in multiple diseases [136,137]. JAK2 is the most intensively investigated member of the JAK family as it is has been shown to play a major role in tumour growth and progression, plus the pathogenic JAK2^V617F^ mutation is present in the majority of patients with myeloproliferative neoplasms (MPNs) [138,139]. The development of JAK2 inhibitors has therefore received significant attention in recent years, including synthesis of the FDA approved JAK1/2 inhibitor ruxolotinib [140]. Additionally proven to be effective against MPNs are HDAC inhibitors, both in isolation as a single agent and when combined with JAK inhibitors [32,141,142]. HDAC inhibitors are capable of decreasing JAK2 expression in MPN cells bearing the JAK2^V617F^ mutation, thus decreasing JAK/STAT signalling and inhibiting tumour growth [32]. Consequently, a multitargeted approach using JAK and HDAC inhibitors provides huge therapeutic benefit.

In 2016, the Dymock group reported compound **19** as the first example of a selective JAK2/HDAC6 dual inhibitor [143]. The JAK2-selective inhibitor pacritinib was fused with vorinostat to afford a series of hybrids incorporating aromatic or nonaromatic linkers of varying lengths and either a monodentate carboxylic acid or bidentate hydroxamic acid ZBG. Synergistic effects of pacritinib in combination with HDAC inhibitors had been observed in various studies, plus its macrocyclic ring was a feature observed in many potent and selective HDAC inhibitors, hence it served as an ideal candidate for incorporation into a multitargeted strategy. Pacritinib also possesses a side chain which protrudes into a solvent exposed region upon inhibitor binding to JAK2, hence it was postulated that equipping the HDAC binding group here would retain JAK2 inhibition and establish HDAC inhibition, with the bulky macrocyclic cap envisioned to achieve some selectivity towards HDAC6 by exploiting the larger lip of the binding pocket. Investigation of enzyme inhibitory activity revealed potent nanomolar JAK2 inhibition and selective inhibition of HDAC6 over HDAC1 for both carboxylic acid and hydroxamic acid hybrids. As expected, due to stronger chelating effects with the zinc ion, the hydroxamic acid-based hybrids were significantly more potent than the carboxylic acid-based equivalents, with 1000-fold greater inhibitory activity and single-digit nanomolar IC_50_ values consistently. Consequently, due to balanced potency towards both JAK2 and HDAC6, the hydroxamic acid dual inhibitors were prioritised for further study. Linker length had a significant effect on HDAC6 inhibition, with inhibitory activity increasing with increased chain length. It was observed that hybrid **19**, comprising an aliphatic linker of six methylene units, was optimal, with an IC_50_ value of 2.1 nM for HDAC6. An approximate 8-fold drop off in potency was observed upon addition of a seventh methylene group (IC_50_ = 15.8 nM). Additionally, **19** demonstrated the greatest inhibition of JAK2 (IC_50_ = 1.4 nM) of all assayed hybrids. When screened against the 11 zinc-dependent HDAC isoforms, compound **19** displayed impressive HDAC6 selectivity (Table 2), with negligible activity against the class-IIa isoforms (HDACs 4,5,7 and 9), >1000-fold selectivity over HDAC3 (IC_50_ = 2.17 µM), >100-fold selectivity over HDACs 1,8 and 11, plus >20-fold selectivity over HDACs 2 and 10. Hybrid **19** was also shown to be >50-fold selective for JAK2 over the other 3 JAK isoforms.

Exploration of antiproliferative activities revealed **19** to be potent across a range of solid and haematological cell lines [143]. In HEL92.1.7 cells expressing the JAK2^V617F^ mutation, Ac-H3 (mediated by HDACs 1,2 and 3) and Ac-tubulin (mediated by HDAC6) levels were compared following treatments with **19** and parent HDAC inhibitor vorinostat. Results revealed compound **19** induced a smaller increase in Ac-H3 levels in comparison to vorinostat and required a 10-fold increase in concentration to achieve a comparable response. In contrast, **19** was 2 to 5-fold more sensitive than vorinostat regarding its Ac-tubulin response, with a significant increase in the level of Ac-tubulin observed even at the lowest concentration tested for **19**, but no increase seen for vorinostat at this concentration. Both sets of data were in-line with compound **19** possessing HDAC6 selectivity. Additional investigation in HEL92.1.7 cells revealed **19** increased phosphorylated JAK2 (p-JAK2) and suppressed STAT5 levels, and hence inhibited the JAK/STAT signalling pathway.

In analogous studies by three independent groups, the hybrids **20**–**22** were also shown to display dual JAK/HDAC inhibition with a degree of selectivity towards HDAC6 [144,145,146]. Alike pacritinib in the synthesis of **19**, each work utilised a JAK (or JAK2-selective) inhibitor containing an aminopyrimidine unit flanked by two hydrophobic aromatic groups—crucial for binding to the hinge region of the JAK active site. Additionally, in the design of each hybrid, the solvent exposed component of the JAK inhibitor was utilised to append the hydroxamic acid binding motif, such that the modification would be well tolerated and conserve JAK inhibition. Continuing their previous work, the Dymock group reported compound **20** which displayed sub-nanomolar inhibition against both JAK2 (IC_50_ = 0.9 nM) and HDAC6 (IC_50_ = 0.1 nM), with a 7000-fold selectivity over HDAC1 (IC_50_ = 774 nM) [144]. An observed significant increase in Ac-tubulin and mild Ac-H3 increase were consistent with HDAC6 selectivity for **20**. Liang et al. reported the dual inhibitor **21**, which demonstrated moderate selectivity for HDAC6 (IC_50_ = 14 nM) over HDAC1 (IC_50_ = 120 nM) and was significantly less potent towards HDAC8 (IC_50_ = 2.47 µM) [145]. Having incorporated a non-selective JAK inhibitor, compound **21** exhibited equivalent non-selective JAK inhibition to its parent JAK inhibitor, with IC_50_ values for JAK1/2/3 between 4–8 nM and 49 nM for TYK2. Interestingly, **21** demonstrated greater antiproliferative activity in HEL cells bearing the JAK2^V617F^ mutation (IC_50_ = 0.09 µM) than the approved inhibitors vorinostat (IC_50_ = 0.65 µM) and ruxolitinib (IC_50_ = 18.6 µM), plus their combination (IC_50_ = 0.30 µM). Huang and co-workers synthesised hybrid **22** through fusion of a JAK2-selective inhibitor with the hydroxamic acid pharmacophore from panobinostat [146]. Compound **22** exhibited potent inhibition of JAK2 (IC_50_ = 8.4 nM) complete with good selectivity for HDAC6 (IC_50_ = 46 nM) over the class-I HDAC isoforms (IC_50_ = 1100, 7472, 234 and 6065 nM for HDACs 1,2,3 and 8, respectively). Further investigation of **22** revealed potent antiproliferative activity toward haematological cell lines as well as proficient in vivo antitumour efficacy over reference inhibitors in an acute myeloid leukaemia (AML) xenograft model [146].

### 4.2. Other HDAC6-Selective Dual Inhibitors

Alongside JAK kinases, there have also been reports of HDAC6-selective dual inhibitors which target other proteins of interest (Figure 8). Heat shock protein 90 (Hsp90) is a molecular chaperone that assists in the refolding of damaged proteins. Synergism has been displayed between Hsp90 inhibitors and HDAC inhibitors, with the latter also shown to inhibit ATP binding and chaperone function of Hsp90, thus, their combination in a multitargeted approach offers significant therapeutic potential. Hybrid **23** was afforded through fusion of vorinostat and 4-isopropyl resorcinol, a crucial structural component present in many Hsp90 inhibitors [147]. Investigation of HDAC inhibition activity revealed **23** to potently inhibit HDAC6 (IC_50_ = 1.15 nM) with >100-fold selectivity over HDACs 1,3 and 8 (inhibited with IC_50_ values of 0.13, 0.16 and 0.28 µM, respectively). Following treatment in HL60 cells, **23** induced a more pronounced increase in Ac-tubulin and noticeably subtler increase in Ac-H3 compared to vorinostat. Moreover, **23** also demonstrated efficient in vitro inhibition of Hsp90 (IC_50_ = 46.3 nM); then, in HL60 cells, it induced degradation of client proteins such as Akt along with upregulation of chaperone protein Hsp70—characteristic markers for Hsp90 inhibition.

A comparable study by He et al. fused vorinostat with the mouse double minute 2 (MDM2) inhibitor nutlin-3 to generate the dual inhibitor **24** [148]. MDM2 is an E3 ubiquitin ligase which functions as the primary negative regulator of the p53 tumour suppressor protein, inducing its degradation via the ubiquitin–proteasome pathway and repressing p53 transcriptional activity [149]. MDM2 is overexpressed in numerous cancers, thus, inhibitors blocking the MDM2-p53 protein–protein interaction to restore p53 activity are an attractive therapeutic strategy [150]. Co-treatment of nutlin-3 and HDAC inhibitors have demonstrated a collaborative effect in the induction of apoptosis, mediated by enhanced p53 levels and p53 hyperacetylation [151,152]. Hybrid **24** demonstrated impressive isoform selectivity for HDAC6 (IC_50_ = 17.5 nM) over the class-I enzymes (IC_50_ = 821, 421, 178 and 1224 nM for HDACs 1, 2, 3 and 8, respectively) along with proficient MDM2 binding affinity (*K*_i_ = 110 nM). Across four solid tumour cell lines, **24** consistently exhibited greater antiproliferative activity than reference inhibitors vorinostat, CI-994 and nutlin-3. A superior in vivo potency was also demonstrated for compound **24**, where following a three-week 100 mg/kg/day treatment in an A549 xenograft model, it achieved a TGI of 65.4%, better than equivalent treatments with vorinostat (57.3%) and nutlin-3 (44.0%) [148].

Preceding the development of the class-I HDAC-targeting PDE5/HDAC dual inhibitor **13**, Rabal et al. also reported the HDAC6-selective example **25** [131]. Alike **13** and **14**, the PDE5 inhibitors sildenafil and vardenafil were employed in the study, equipped via their solvent exposed motif with various aromatic linkers bearing a hydroxamic acid ZBG to afford a series of hybrids. Thiophene-containing sildenafil analogue **25** emerged as the most potent compound against HDAC6 (IC_50_ = 15 nM) and achieved balanced PDE5 inhibition (IC_50_ = 11 nM). Moreover, **25** exhibited impressive selectivity for HDAC6, with 20-fold selectivity over HDAC1 (IC_50_ = 345 nM), >100-fold selectivity over HDAC2 and HDAC3 (IC_50_ = 3720 and 2090 nM), and no activity (IC_50_ = >10 µM) against other class-I (HDAC8) and class-IIa (HDACs 4,5 and 9) isoforms. In SH-SY5Y neuroblastoma cells, 100 nM treatment with **25** induced a 10.5-fold increase in Ac-tubulin levels (over basal levels) and a 1.8-fold increase in AcH3K9 levels, further confirming selectivity towards HDAC6. These responses were more potent and selective than the HDAC6-selective inhibitor tubastatin A, which at 100 nM induced a 5.5-fold increase in Ac-tubulin levels and a 1.3-fold increase in AcH3K9 levels. Further investigation revealed **25** increased CREB phosphorylation levels, plus in Tg2576 neurons, reduced levels of the AD-related markers hAPP (human amyloid precursor protein) and pTau (phosphorylated tau protein) by 55% and 30%, respectively, following 100 nM treatment for two days [131].

## 5. HDAC Complex-Selective Dual Inhibitors

Much of the HDAC inhibitor development programmes to date have focused on isoform-selective inhibitors, however, for targeting class-I HDACs, there is an additional avenue to exploit. With the exception of HDAC8, the class-I HDACs (HDACs 1–3) are recruited to multi-protein complexes via direct interaction with one of at least 17 co-repressor proteins [153]. HDAC3 uniquely exists as the catalytic subunit for the SMRT/NCoR (silencing mediator for retinoid and thyroid hormone receptor/nuclear receptor co-repressor) [154,155] complex, whereas HDAC1 and HDAC2 are interchangeably recruited to 6 separate complexes—CoREST (co-repressor of REST) [156,157], MiDAC (mitotic deacetylase) [158,159], NuRD (nucleosome remodelling and deacetylase) [160,161], SIN3 (switch-independent 3) [162,163], MIER (mesoderm induction early response) [164,165], and RERE (arginine–glutamic acid dipeptide repeats) [166]. Like individual HDAC isoforms, specific HDAC epigenetic regulatory complexes have been implicated with certain diseases, thus complex-selective inhibitors could serve as important therapeutic agents [52]. Each of these complexes has a unique function in cells [167], and therefore the expectation is that “complex-specific” inhibition will replicate some of positive effects of generic HDAC inhibitors, but with reduced side-effects. In the case of HDACs 1 and 2, due to an 86% sequence homology, targeting the respective enzymes individually has proved very challenging. However, as the two enzymes reside in multiple complexes, an approach targeting individual complexes may be more practical and clinically useful. One method of achieving this selectivity is through the development of dual inhibitors which target the HDAC and one of the co-repressor proteins within a certain complex. This has been demonstrated in recent years for the dual inhibition of the CoREST complex but has the potential to be applied for any of the other complexes.

**Figure 9:**
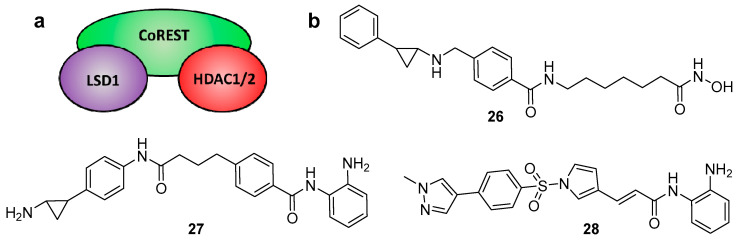
(**a**) Schematic representation of the CoREST corepressor complex. (**b**) Reported examples of dual HDAC-LSD1 inhibitors.

The CoREST epigenetic complex consists of HDAC1/2 and the monoamine oxidase LSD1 (lysine-specific demethylase 1A), linked together via the scaffold protein CoREST (Figure 9a). Like HDACs, LSD1 is a recognised epigenetic eraser and drug target that plays a critical role in the modification of histones [168]. When recruited to the CoREST complex, LSD1 catalyses the removal of activating mono- and di-methyl marks on nucleosomal H3K4 residues, resulting in transcriptional repression [169,170]. Studies have revealed a key functional interplay (or “crosstalk”) between HDAC and LSD1 within the complex, where the enzymes mutually influence each other’s activity via the CoREST protein [171,172]. HDAC inhibition has been shown to attenuate LSD1 activity [171,173], plus, synergistic effects of combined HDAC and LSD1 inhibition have been reported [174,175,176,177]. Consequently, there is a proven rationale for the development of dual HDAC/LSD1 inhibitors to selectively target the CoREST complex and deliver a combined epigenetic inactivation of gene expression (Figure 9b).

In 2017, Duan et al. reported a series of dual HDAC and LSD1 inhibitors, via conjugating the LSD1 inhibitor tranylcypromine to various *o*-aminoanilide and hydroxamic acid-based HDAC inhibitors [178]. The best of these dual inhibitors, **26**, utilised vorinostat as the HDAC inhibitor and displayed potent IC_50_ values of 15 nM and 23 nM against HDAC 1 and 2, respectively, plus inhibition against LSD1 with an IC_50_ of 1.20 μM. The levels of acetylation and methylation of certain histone biomarkers displayed dose-dependent increases with **26**, further confirming dual inhibition of the two enzymes. Additionally, **26** was shown to display stronger antiproliferative activities than vorinostat alone against multiple cancer cell lines.

In an elegant study by Kalin et al., fusion of the class-I-selective HDAC inhibitor entinostat and a tranylcypromine analogue of the LSD1 inhibitor bizine afforded the dual inhibitor **27**, also known as corin [179]. LSD1 and pan-HDAC assays revealed **27** be a potent dual inhibitor, with greater than 100-fold selectivity for HDACs 1–3 (IC_50_ values < 200 nM) over HDACs 4–9 (IC_50_ values > 30 µM). Inhibition properties were then investigated in a more meaningful scenario against purified CoREST complex itself, revealing **27** to be as effective as its individual component inhibitors at blocking HDAC1 and LSD1 activity. A crucial finding arose from analysis of the inhibition kinetics, wherein **27** displayed sustained and selective inhibition of the CoREST complex owing to its dual targeted action and irreversible binding of the LSD1 inhibitor component. Significantly reduced CoREST complex HDAC1 activity was observed 6 h after a 30 min exposure to **27**, whereas analogous treatment with entinostat or entinostat and bizine resulted in very little loss in activity after this time. Additionally, jump dilution assays revealed corin to exhibit near irreversible inhibition of CoREST complex HDAC1 activity compared to the constituent HDAC and LSD1 inhibitors. Upon testing **27** further, it was also found to possess enhanced antiproliferative effects and antitumour properties over entinostat.

Domatinostat (**28**) was first reported as a class-I-selective HDAC inhibitor with promising antitumour activity against pre-clinical colorectal cancer models [180]. However subsequent work has revealed that it also inhibits LSD1, thus functioning as a dual inhibitor [181,182]. It is currently in clinical trials in combination with checkpoint inhibitors (which enhance the body’s immune response for treatment) for advanced-stage Merkel cell carcinoma.

## 6. HDAC Proteolysis-Targeting Chimeras (PROTACs)

A novel approach utilising bifunctional HDAC inhibitors has been in the development of proteolysis-targeting chimeras (PROTACs) to target HDAC enzymes for induced protein degradation. These compounds comprise of three components: a HDAC inhibitor, an E3 ligase ligand, and a linker which couples these two moieties together. In binding to its respective targets, the PROTAC recruits the HDAC to an E3 ligase, forming a ternary complex. Subsequent polyubiquitination of the HDAC enzyme occurs, which tags it for selective degradation via the proteasome. First reported in 2001 by Sakamoto et al., the PROTAC technology has received significant interest in recent years, particularly as a strategy to treat difficult-to-drug protein targets [183,184]. It has experienced a series of advancements in its design along the way, with the introduction of all small molecule-based PROTACs as well as incorporation of new E3 ligase ligands, affording a wide range of potent protein degraders [185,186,187,188]. In addition to demonstrating therapeutic effects, PROTACs have also proved important tools for biological discovery [189]. A key feature to the PROTAC mode of action is the ability to act in a catalytic manner. By incorporating a reversible target protein inhibitor into the design, a PROTAC can dissociate from the ternary complex following the polyubiquitination process, then bind to another target protein thereby enabling repeated cycles of induced protein degradation.

An HDAC enzyme degradation approach offers a number of potential advantages over inhibition regarding their use as future therapeutic agents. Firstly, PROTACs could enable reduced dosing and hence lower systemic drug exposure due to their catalytic mode of action and reduced occupancy level requirement. This may result in significantly reduced side effects in comparison to HDAC inhibitors, which require stoichiometric drug binding for modulation of protein function. Secondly, PROTACs offer the potential of a prolonged duration of action, as restoration of HDAC function will require re-synthesis of the enzyme. PROTACs are capable of multiple rounds of degradation, so following knockdown of HDAC levels, they can be envisaged to maintain low HDAC levels. Finally, alike the benefits of dual inhibitors over mono inhibitors, HDAC PROTACs provide another strategy for enhancing HDAC isoform or complex selectivity, as well as overcoming drug resistance.

**Figure 10:**
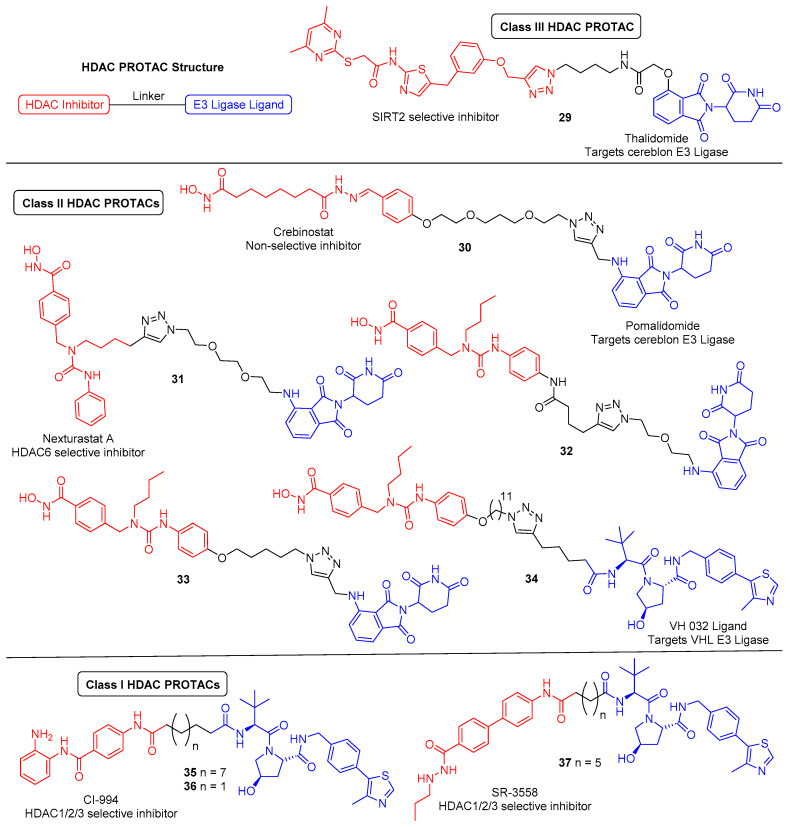
HDAC-targeting proteolysis-targeting chimeras (PROTACs). PROTAC components: HDAC inhibitor (red), small molecule E3 ligase ligand (blue), linker (black).

In light of discoveries over the last couple of years, there are now a variety of HDAC-targeting PROTACs that have been developed, capable of degrading class-I, -II and -III HDACs (Figure 10). The first report of a HDAC-targeting PROTAC was in 2018 by Schiedel et al., who synthesised a SIRT2-selective degrader [190]. This was also the first evidence of the PROTAC technology being used to target an epigenetic eraser protein. As previously discussed, sirtuins (SIRT1-7) make up the class-III HDACs and belong to a different family to the class-I,-II and -IV HDACs, as they require a NAD^+^ cofactor for activity instead of Zn^2+^. The group had recently developed triazole-based SIRT2-selective inhibitors and already utilised these in synthesising biotinylated SIRT2 probes using Cu(I)-catalysed cycloaddition (“click”) chemistry [191]. The structural features from this work provided a rationale for the design of SIRT2 PROTACs, as the previously used alkynylated SIRT2 inhibitor was this time “clicked” to a cereblon E3 ligase ligand (thalidomide) equipped with a terminal azide handle. Docking of the anticipated ternary complex was used to guide choice of linker length, and the resulting PROTAC (**29**) demonstrated SIRT2 degradation of up to 90% at 5 μM in HeLa cells. Time course studies found that maximum effect was observed after 2 h of treatment.

The first zinc-dependent HDAC to be successfully degraded via PROTACs was the class-II isoform HDAC6. Yang and co-workers conjugated the non-selective HDAC inhibitor crebinostat to the cereblon E3 ligase ligand pomalidomide via a polyethylene glycol (PEG) linker to afford **30** [192]. In multiple myeloma MM.1S cells, **30** achieved maximum effect and almost complete HDAC6 knockdown at 80 nM or higher concentrations, with no significant change in the protein levels of HDAC 1,2 or 4 under equivalent conditions. Therefore. despite incorporating a pan HDAC inhibitor, **30** demonstrated an impressive degree of isoform-selective degradation. The formation of a stable ternary complex has been shown to be a vital feature in order to achieve degradation of the target protein. This work suggests that **30** is only capable of forming a stable ternary complex with the cereblon E3 ligase and HDAC6, not any of the other HDACs, thereby providing the observed isoform selectivity. Although not degrading other HDAC enzymes, increased Ac-H3 levels were observed following treatment with **30**, demonstrating that it still functioned as an inhibitor to other HDACs.

Following discovery of **30**, there has since been a flurry of other HDAC6 degraders reported over the last couple of years by the Rao and Tang groups (PROTACs **31**–**34**). All of these PROTACs have incorporated the HDAC6-selective inhibitor Nexturastat A and utilised “click” chemistry to conjugate the inhibitor to the E3 ligase ligand. A co-crystal structure of HDAC6 with Nexturastat A revealed that it binds via a y-shaped conformation, with the hydroxamic acid zinc binding group positioned inside the binding pocket for chelation to the zinc ion, and both the terminal benzene ring and aliphatic carbon chain protruding from the pocket, interacting with the isoform-specific hydrophobic surface rim of HDAC6 [133,193]. The latter surface exposed components thus served as ideal handles to build out from. In successive works, the Rao group investigated both such positions, tethering pomalidomide via PEG linkers to both the aliphatic chain and benzene ring of Nexturastat A, synthesising two sets of degraders [193,194]. Overall, they found that functionalising from both locations of Nexturastat A produced HDAC6-selective degraders. The most potent PROTAC in each case (**31** and **32**) displayed comparable activity and effectively induced selective HDAC6 degradation at 100 nM across different cell lines.

During this time, Wu et al. also “clicked” pomalidomide to the benzene ring of Nexturastat A, here using varying length alkyl linkers either side of the triazole ring to afford a range of potent and selective degraders [195]. The most potent PROTAC, **33**, had a DC_50_ (degradation concentration to achieving half-maximal degradation) of 1.64 nM and could induce its maximal degradation (D_max_) of 86% at as low as 30 nM. Highlighted in this work is also the additional response caused by the incorporation of pomalidomide, which, along with thalidomide, makes up part of a class of drugs known as immunomodulatory imide drugs (IMiDs). These molecules, upon binding cereblon, can induce degradation of neo-substrates, most notably the Ikaros zinc finger (IKZF) proteins. Compound **33** was shown to induce IKZF1/3 degradation in addition to HDAC6 degradation, which resulted in promising antimyeloma activity due to synergistic effects. Although useful in this example, the side effects caused by IMiD containing PROTACs have been questioned to limit their utility. In a follow-up paper, the Tang group recently developed a related HDAC6-selective PROTAC, **34**, now incorporating a different E3 ligase ligand which targets the Von Hippel–Lindau (VHL) E3 ubiquitin ligase [196]. Interestingly, a much longer linker length was required for efficient levels of degradation in comparison to the cereblon-based degraders. Compound **34** maintained potent HDAC degradation (DC_50_ = 7.1 nM, D_max_ = 90%) and, as expected, caused no induced degradation of IKZF1/3 proteins.

**Figure 11:**
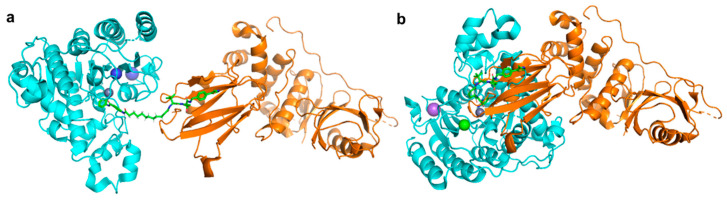
(**a**) With twelve-carbon linker **35** HDAC2 (cyan, PDB 4LY1 [111]) and Von Hippel-Lindau (VHL) E3-ligase (orange, PDB 4W9H [198]) are brought into close proximity. (**b**) With six-carbon linker **36,** unfavourable direct protein-protein overlap and steric clash occur.

Unlike the class-II, -III and -IV HDACs, the class-I HDACs are nucleus localised and exist as part of much larger multiprotein corepressor complexes in vivo. Recently, we reported the first example of a class-I HDAC-targeting PROTAC, which demonstrated degradation of HDACs 1,2 and 3 [197]. The class-I *o*-aminoanilide HDAC inhibitor CI-994 was conjugated to the VHL ligand via a twelve-carbon alkyl linker to synthesise PROTAC **35**. After 24 h treatment in HCT116 human colon cancer cells, **35** achieved almost complete degradation of HDACs 1 and 2 at 10 µM, whilst HDAC3 levels were also decreased. At least 50% degradation was observed for HDACs 1,2 and 3 at 1 µM. Linker lengths incorporating six-carbon linkers with either VHL (**36**) or thalidomide failed to degrade class-I HDACs in cells despite retaining sub micromolar HDAC inhibition in vitro. Using the HDAC2 crystal structure with a directly analogous *o*-aminoanilide inhibitor bound (PDB 4LY1) in the HDAC active site, and the pVHL:EloB:EloC complex with the VHL E3-ligase ligand bound (PDB 4W9H), these proteins were modelled by tethering their respective ligands together via a twelve carbon linker and six carbon linker, recreating **35** and **36** from our study (Figure 11). It can be seen with the twelve-carbon linker, **35**, VHL and HDAC2 are brought into close proximity with no direct steric clash or protein–protein overlap, unlike the six-carbon linker, **36**, suggesting that if **36** is cell permeable, it may be unable to form the necessary ternary complex required for polyubiquitination and degradation.

Following the discovery of **35**, Xiao et al. reported the HDAC3 specific PROTAC **37**, which like **35** is a VHL-recruiting degrader and utilises an alkyl linker in its design [199]. The incorporated HDAC inhibitor SR-3558 was previously identified by the group as being class-I selective and features a novel benzoylhydrazide ZBG [200]. In MDA-MB-468 cells, **37** selectively induced HDAC3 degradation with a DC_50_ concentration of 42 nM following a 14 h treatment, with little to no change in HDAC 1,2 or 6 protein levels. Washout assays revealed the impact of **37** on HDAC3 protein levels was long-lasting and reversible, requiring over 12 h to restore to pre-treatment levels. With an enhanced selectivity profile observed for **37** compared to the parent inhibitor, future research in this area may lead to the development of HDAC complex selective or other class-I HDAC isoform-selective PROTACs.

## 7. Conclusions and Future Outlook

The FDA approval of five HDAC inhibitors has firmly established the importance of HDACs as a therapeutically relevant target and paved the way for the development of new inhibitor designs to achieve improved efficacy and selectivity profiles. HDACs are associated with various multifactorial diseases, hence, combining HDAC inhibition with that of another disease-related target has emerged as a valuable approach to deliver a more directed and sustained treatment over single-targeting agents. This polypharmacological treatment has been successfully achieved using bifunctional HDAC therapeutics—single molecule, dual-targeting agents comprising a HDAC inhibitor conjugated to another specificity-targeting moiety. In many cases, dual HDAC inhibitors have been developed following observed synergy between HDAC inhibitors in combinatorial therapy with other drugs. The HDAC inhibitor pharmacophore can tolerate extensive cap modification and so is very amenable to the synthesis of such hybrids. Already, an extensive list of targets has now been successfully inhibited via dual HDAC inhibitors, complete with impressive potency and high levels of HDAC isoform or class selectivity. This list is set to expand in the years ahead.

The class-I HDACs exist as part of multi-protein complexes in vivo, presenting another factor to consider for inhibition along with the high homology present between the isoforms. Dual HDAC inhibitors offer the potential to target the HDAC and one of its protein partners within a specific complex, providing a novel strategy to achieve complex selectivity. This approach has already been achieved in targeting the CoREST complex, concurrently inhibiting both HDAC and LSD1, but has the potential to be extended to other HDAC co-repressor complexes.

PROTACs are an interesting deviation for bifunctional HDAC modulators, offering an alternative strategy for achieving enhanced selectivity over inhibition alone. Whilst still in its infancy, recent findings are delivering highly selective and potent HDAC degraders, thus sparking a new area for discovery of potential future therapeutics. Development of complex-selective HDAC PROTACs would be an exciting prospect as a new means to treat complex-specific diseases.

## Figures and Tables

**Table 1:** Isoform selectivity of hybrids **7** and **8** against HDACs and CDKs.

Hybrid	IC_50_ (nM)							
	HDAC1	HDAC2	HDAC3	HDAC6	HDAC8	CDK1	CDK2	CDK4,6,7
**7**	240	0.24	>1000	>1000	>1000	12.58	0.56	>1000
**8**	6.4	0.25	45	>1000	>1000	8.63	0.30	>1000

**Table 2:** Isoform selectivity profile of hybrid **19** against class-I,-II and -IV HDACs.

HDAC Class HDAC Isoform	I				IIa	IIb		IV
	1	2	3	8	4,5,7,9	6	10	11
IC_50_ (nM) or % inhibition	222	49	2170	740	<20% at 10 µM	2.1	80	930
